# Correction: Midwives’ and obstetricians’ perspectives about pregnancy related weight management in Ethiopia: A qualitative study

**DOI:** 10.1371/journal.pone.0247720

**Published:** 2021-02-23

**Authors:** Fekede Asefa, Allison Cummins, Yadeta Dessie, Maralyn Foureur, Andrew Hayen

The fourth author’s name is spelled incorrectly. The correct spelling is: Maralyn Foureur. The correct citation is: 1. Asefa F, Cummins A, Dessie Y, Foureur M, Hayen A (2020) Midwives’ and obstetricians’ perspectives about pregnancy related weight management in Ethiopia: A qualitative study. PLoS ONE 15(12): e0244221. https://doi.org/10.1371/journal.pone.0244221
