# Supplementary material for: Midwives’ and obstetricians’ perspectives about pregnancy related weight management in Ethiopia: A qualitative study
Source: PLoS One. 2020 Dec 17;15(12):e0244221. doi: 10.1371/journal.pone.0244221 (PMC7746277; doi:10.1371/journal.pone.0244221)
Supplement: S1 Table — (DOCX) [file pone.0244221.s001.docx]

#### S1 Table. The topic guide for interviews with obstetricians and midwives, Addis Ababa, Ethiopia, 2019

| English version | |
| --- | --- |
|  | How do you explain GWG? |
|  | Would you please tell me how much weight pregnant women are recommended to gain?  **Probe:** would you please tell me in detail according to women’s pre-pregnancy weight: for underweight, normal weight, overweight and obese women. |
|  | Would please tell me about GWG guideline you are aware of (if any)  **Probe:** Do you have a GWG guideline?  If yes, would you tell me about it? What are the contents of the guideline?  If there is no guideline, to what extent you need the guideline? What type of information you want to be included to the guideline?  Have you ever heard of or seen IOM guidelines for weight gain?  If yes, would you tell me details of the IOM GWG recommendations? |
|  | How do you manage GWG?  **Probe**: What type of information you provide for pregnant women? Type of advice for pregnant women about GWG, about nutrition, about physical activities…  Do you specifically tell pregnant women in detail the amount of weight they already gained and the amount they are recommended to gain? If no, why not?  Do you encourage women to follow their own weight? If no, why not? |
|  | How do feel discussing GWG with women?  **Probe:** how sensitive it is?  Level of priority? Why a priority or why not a priority?  How much are you confident in providing advices on GWG and nutrition? What should be done next? |
|  | Would you please tell me your view on post-partum weight management?  **Probe:** what women’s postpartum weight management looks like? Would you tell me from you observation?  What advise you provide for pregnant women during ANC follow up regarding postpartum weight management? If there is no advise, why not? |
| **Amharic version (Local language)** | |
|  | ሴቶች በእርግዝና ጊዜያቸው የሚጨምሩትን ክብደት እንዴት ትግልጻለህ/ለሽ? |
|  | እርጉዝ ሴቶች ምን ያክል ክብደት እንደምሩ ይመክራል?  **ማዉጣጫ፡** ሴቶች ከእርግዝና በፊት ካላቸዉ ክብደት ጋር በማያያዝ በስፋት ብትንግረኝ/ሪኝ፤ ማለትም አንደር፟ዌይት፤ ኖርማልዌይት፤ ኦቨርዌይት እና ኦቤስ |
|  | እርጉዝ ሴቶች የሚጨምሩትን ክብደት ለመቆጣጠር የሚረዳ መመሪያ(ጋይድላይን) በተመለከተ ብትንግርኘ/ኚን (የምታዉቂዉ/ቀዉ ካለ)  **ማዉጣጫ፡**እርጉዝ ሴቶች የሚጨምሩትን ክብደት ለመቆጣጣር የሚረዳ መመሪያ(ጋይድላይን) አላችሁ? ካለ በስፋተ ቢነግረኝ/ሪኝ፤ የመመሪያዉን ይዘት ቢታብራራዉ/ሪዉ?  መመሪያዉ ከለለ መመሪያዉን ምን ያክል ትፈልጋለህ/ጊያለሽ? መመሪያው ምን አይነት መረጃ እንድያካትት ትፈልጋለህ/ጊያለሽ?  አይ ኦ ም የክብደት መቆጣጠርያ መመሪያ(ጋይድላይን) አይተህ/ሽ ወይም ሰምተህ/ሽ ታዉቃለህ/ቂያልሽ?  ካውቅሽ/ህ ይዘቱን ብታብራራ/ሪ |
|  | በእርግዝና ጊዜ የሴቶችን ክብደት ለመቶጣጠር ምን ታደርጋለህ/ለሽ?  **ማዉጣጫ፡** ለእርጉዝ ሴቶች ምን አይነት መረጃ/ምክር ትሰጭያልሽ/ጣለህ? በክብደት መጨመር ወይም መቆጣጠር፤ ስን፟ምግብ፤ የአካል እንቅስቃሴ ዙሪያ የሚሰጥ ምክር  ለእርጉዝ ሴቶች በተለየ መልኩ ምን ያክል ክብደት እንደጨመሩ ወይም መጨመር እንዳለባቸዉ ትናገራለህ/ሪያለሽ? ካልሆን ለምን?  እርጉዝ ሴቶች ክብደታቸዉን እንዲከታተሉ ታበረታታለህ/ታበረታቺያለሽ? ካልሆነ ለምን? |
|  | ስለሴቶች ክብደት መጨመር ስታወራ/ሪ ምን አይነት ስሜት ይሰማሃል/ሻል?  **ማዉጣጫ፡** ምን ያክል ስሜት ነክ ነው ወይም ያሳቅቃል?  እርጉዝ ሴቶችን ስለክብደት መጨመር ለመምከር ምን ያክል ቅድሚያ ትሰጣለህ/ጭያለሽ? ለምን?  እረጉዝ ሴቶችን በክብደት መጨመር ወይም ስነ፟ምብ ዙሪያ ለመምከር ብቁ ነኝ ብለህ/ሽ ታስባለህ/ቢያለሽ? ካልሆንክ/ሽ በቀጣይ ምን መደረግ አለበት? |
|  | ከወሊድ በህዋላ የክብደት መቆጣጠር ሂደት ላይ ያለሽን/ህን ምልከታ ብትንግሪኝ/ረኝ?  **ማዉጣጫ፡** ከወሊድ በህዋላ የሴቶች የክብደት ቁጥጥር ሂደት ምን ይመስላል? ከሽካባቢዉ ወይም ብስራ ሁኔታ ከምታየዉ /ይው ብታብራራ/ሪ  ከወሊድ በህዋላ የሴቶች የክብደት ቁጥጥር ዙሪያ ለእጉዝ ሴቶችምን አይነት ምክር ት ሰጭያልሽ/ስጣለህ? ካልሰጠህ/ሽ ለምን? |
